# Assessment of the Dietary Intake of Schoolchildren in South Africa: 15 Years after the First National Study

**DOI:** 10.3390/nu8080509

**Published:** 2016-08-19

**Authors:** Nelia Steyn, Gabriel Eksteen, Marjanne Senekal

**Affiliations:** 1 Division of Human Nutrition, Department of Human Biology, University of Cape Town, Cape Town 8000, South Africa; gabriel@heartfoundation.co.za (G.E.); marjanne.senekal@uct.ac.za (M.S.); 2 South African Heart and Stroke Foundation, Cape Town 8000, South Africa

**Keywords:** schoolchildren, dietary intake, fortification, South Africa, micronutrients

## Abstract

There has not been a national dietary study in children in South Africa since 1999. Fortification of flour and maize meal became mandatory in October 2003 to address micronutrient deficiencies found in the national study in 1999. The purpose of this review was to identify studies done after 1999 in schoolchildren, 6–15 years old, in order to determine whether dietary intakes reflected improvements in micronutrients, namely: iron, zinc, vitamin A, folate, thiamine, riboflavin, vitamin B6, and niacin. An electronic and hand search was done to identify all studies complying with relevant inclusion criteria. The search yielded 10 studies. Overall, there is a paucity of dietary studies which have included the fortified nutrients; only four, of which only one, reported on all micronutrients; making it difficult to determine whether fortification has improved the micronutrient intake of schoolchildren. This is further complicated by the fact that different dietary methods were used and that studies were only done in three of the nine provinces and thus are not generalizable. The results of these studies clearly point to the importance of doing a national study on the dietary intake of schoolchildren in order to confirm the outcomes of the fortification process.

## 1. Introduction

Schoolchildren are nutritionally very vulnerable both at junior and at high school levels. This is explained amongst others by the fact that they spend a considerable amount of time out of the home, while it is also a period of rapid growth, particularly during adolescence [1,2]. Children and adolescents are also well known to make poor food choices [3]. Most schools in South Africa have a tuck-shop (food store) and these frequently sell items which are energy dense (high in fat and sugar) and low in micronutrients [4]. Examples include donuts, crisps, fat cakes (fried dough balls), and carbonated sweet beverages [5]. This is a situation which could be contributing to the increase in childhood obesity and micronutrient deficiencies

However, it should also be noted that, in South Africa, a large percentage of the population are economically challenged since the unemployment rate is 29% [6]. Hence a large number of children live in food insecure homes and are themselves vulnerable to undernutrition and micronutrient deficiencies. Consequently, schoolchildren are exposed to both over and undernutrition, which emphasizes the importance of surveillance data on dietary intake [7].

The first, and only, national survey on dietary intake data in children (National Food Consumption Survey-NFCS) in South Africa took place in 1999 and has been reported on extensively [8,9,10,11,12,13]. The survey population comprised all children aged 1–9 years. A total of 156 Enumerator Areas were included in the study of which 82 were urban and 74 were rural. A total of 3120 children aged 1–9 years were included in the survey. The dietary assessment tools used were the 24 h recall (24 HR) and a quantified food frequency questionnaire (QFFQ). These were completed by means of a personal interview with the mother [8,9,10,11,12,13].

The NFCS showed that a large proportion of children were at risk of deficiency in energy and numerous micronutrients, including calcium, iron, zinc, vitamin A, vitamin C, riboflavin, niacin, and vitamin B6. These micronutrients were those where the most children had intakes less than 67% of the recommended dietary allowances (RDAs) [8]. Since the estimated average intakes (EARs) [14] are now used, and are lower than 67% of the RDAs, it may be that some of the risks of deficiencies were overestimated. The five most commonly eaten foods in the NFCS were maize, white sugar, tea, whole milk, and brown bread. While it is encouraging to see that milk was one of the most commonly consumed food items, the portions were small and the milk was frequently used in tea or coffee. In the NFCS, tea was the third most popular item consumed by children after maize porridge [8]. However, the average portion size of milk per day was very small (74.6 g). The majority of the diet comprised sources of carbohydrate-rich items and the intake of fruit and vegetables was very low [8,9,10,11,12,13].

The poor quality of the diet was reflected by the high prevalence of stunting and underweight. The prevalence of stunting was highest in Free State (39.8%), North West (31.9%), Northern Cape (30%), and Mpumalanga (29.1%), with the national prevalence being 25.5%. The prevalence of underweight was highest in Northern Cape (27.1%), Free State (20.4%), and North West, (18.6%) with the national prevalence being 12.4%. The prevalence of stunting in 7–9 years old was 13% and the prevalence of underweight in this age group was 7.7%. The prevalence of overweight in 7–9 years old children was highest in Mpumalanga (20.7%), Northern Cape (11.8%), and Gauteng (8.0%) with the national prevalence being 6.1% (weight for height >2 SD) [8]. Studies prior to 1999 had also demonstrated vitamin A and iron deficiencies in blood samples of pre-schoolchildren [15]. 

In order to combat malnutrition in South Africa, the government introduced numerous initiatives, including national food fortification, iodization of salt, and a national primary school feeding program (PSNP) [16]. Other initiatives have included iron, folate, and vitamin A supplements to pregnant women and babies [16]. From 2004, maize meal and wheat flour have been enriched with iron, zinc, thiamin, riboflavin, niacin, vitamin B6, folate, and vitamin A since these were the micronutrients most commonly deficient in the diet as assessed by the NFCS [17]. The school feeding program provides a mid-day school meal comprising a carbohydrate, protein, and fruit or vegetable to schoolchildren in areas classified as being of low socio-economic status [16]. The expectation now is that these initiatives should have improved the dietary intake and nutritional status of schoolchildren.

Some evidence of improvement comes from the South African National Health and Nutrition Survey (SANHANES) which took place in 2012 [7]. This study measured many parameters of health and nutrition in adults and children including nutritional weight status, hunger and dietary diversity by means of a 24 HR which was not quantified; hence quantified data on macro- and micro-nutrient content could not be assessed. The findings indicate that stunting decreased from 13.0% in 7 to 9 years-old in 1999 [8] to 10% in boys and 8.7% in girls in 2012 [7]. There does not seem to have been a change in underweight. Furthermore, overweight and obesity in 7 to 9 years old was 6.1% in the NFCS in 1999 and increased to 7.2% in boys and 16.4% in girls in 2012. Thus the malnutrition problem seems to have shifted to include concerns about the increasing prevalence of overweight/obesity. However the SANHANES data should be interpreted cautiously since the ages differ slightly (6 to 9 vs. 7 to 9) and white and Asian children were not included in SANHANES data.

Since there has not been a national dietary intake survey since 1999, it is not possible to know how dietary intake and specifically intake of the fortified nutrients has changed and contributed to shifts in nutrition indicators in the past 15 years in schoolchildren. However, a number of smaller, localized dietary intake surveys have taken place in schoolchildren. This review is aimed at utilizing the dietary intake results of studies which have taken place in schoolchildren (6 to 15 years) between 2000 and 2015, to shed light on possible changes which have occurred as a result of the dietary interventions rolled out by the government.

## 2. Materials and Methods 

This review employed electronic and manual searching of peer reviewed and grey literature, as well as electronic data sets of national and local or unpublished studies done on the dietary intake of South African schoolchildren since 2000. 

The databases searched were MEDLINE, Academic Search Premier, Africa Wide studies, CINAHL, ERIC, Health Source (Nursing Edition)—all via EBSCOHOST; PubMed; Web of Science; ScienceDirect; Scopus (EMBASE); and Google Scholar (2013+). Databases were searched for publications containing the terms “dietary intake”, “food intake”, “nutrient intake”, “caloric/energy intake”, “food habits”, “dietary trends”, “diet/nutrition survey”, “food consumption”, “diet/nutrient assessment”, and “nutritional status” within the keywords, abstract or title. The search was limited to studies that contained the terms “South Africa” or “South African” in either the title, abstract or keywords, and publications from 2000 onwards. Furthermore, the South African Journal of Clinical Nutrition was hand-searched from the year 2000 for dietary studies on children and South African University websites were searched for theses.

Studies were included in the review according to the following inclusion criteria:

* Dietary studies which used one of the following methods: 24 h recall, food frequency, weighed dietary record, dietary history to record children’s nutrient intakes, and/or foods consumed.
* Participants in the studies were between 6 and 15 years old.
* Studies included in the review had at least 30 participants per group.
* The study results included energy and macro/micro nutrient intakes or foods commonly consumed.

Studies were excluded for the following reasons:

* Participants of the studies had a specific disease condition e.g., diabetes or HIV/AIDS.
* Participants were disabled.

## 3. Results

Since 2000, there have been 10 studies [18,19,20,21,22,23,24,25,26,27] on children aged 6 to 15 years (Table 1). Seven of the studies used a 24 HR of which one study used two 24 HRs and one used three 24 HRs. Three studies used a QFFQ to obtain dietary data. Three studies [18,19,20] were published before fortification was introduced in 2004; three studies were published after 2004, with data collection for two of these studies having taken place before 2004 [21,22] and for one after fortification was introduced [23]. The fortification nutrients were, however, not included in the latter study. Four studies were published after fortification was introduced, and the fortified nutrients were included [24,25,26,27]. In South Africa, dietary data is analyzed using the Medical Research Council dietary data software FoodFinder. To date, the fortified nutrients have not been added to maize and flour products in the food composition data base and have to be added separately on the FoodFinder program. The latter four studies reported doing this. However, unfortunately, these four studies do not all report on all the micronutrients. In order to compare data with the estimated average requirements (EARs) the values for the 9–13 years old age group were used since this was the most common age category within which the studies age categories fell. 

All the studies which reported mean energy intakes found that the values were below the estimated energy requirement (EER) based on an active physical activity level. None of the studies reported mean protein intakes below the EAR values for age. 

**Figure 1:**
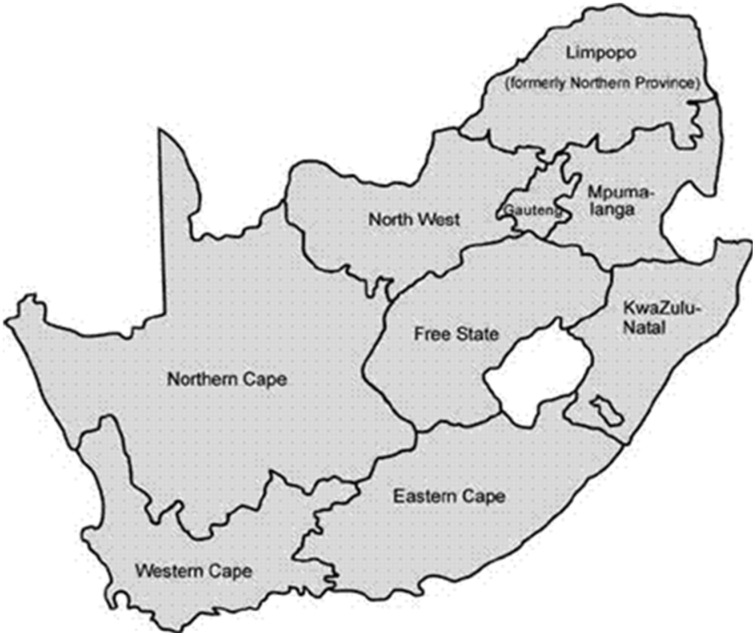
Map of South Africa showing provinces.

**Figure 2:**
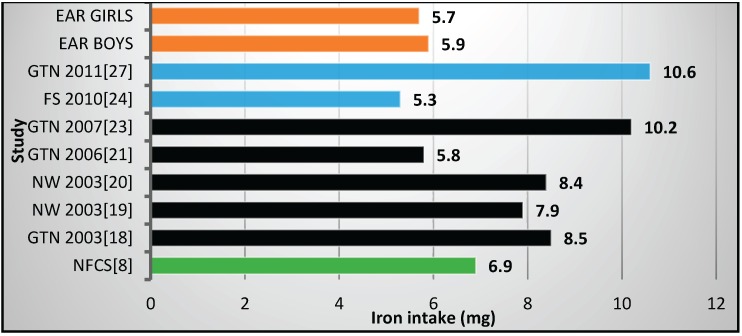
Mean iron intake in studies on schoolchildren in South Africa between 2000 and 2015 with the blue bars representing two studies which included fortification values. EAR: estimated average intake; GTN: Gauteng; FS: Free State; NW: North West; NFCS: National Food Consumption Survey.

Only one study in the Free State Province [24] (Figure 1 map) reported mean iron values below the EAR values (Table 2, Figure 2). This study included fortification values [24]. Even the studies which did not include fortification had mean values above the EARs. It is interesting to note that the NFCS had a mean iron intake value above the EARs.

**Figure 3:**
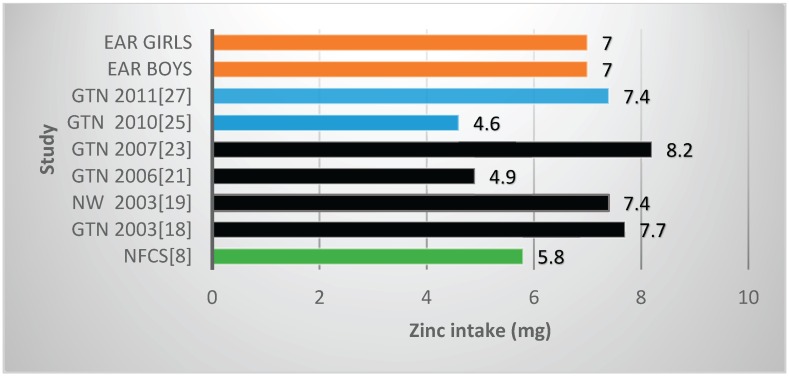
Mean zinc intake in studies on schoolchildren in South Africa between 2000 and 2015 with the blue bars representing two studies which included fortification values. EAR: estimated average intake; GTN: Gauteng; NW: North West; NFCS: National Food Consumption Survey.

**Figure 4:**
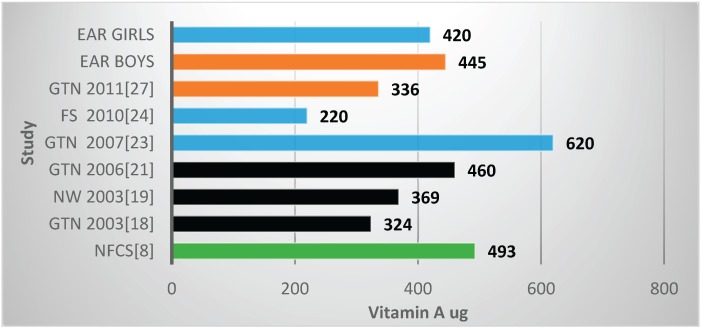
Mean vitamin A intake in studies on schoolchildren in South Africa between 2000 and 2015 with the blue bars representing two studies which included fortification values. EAR: estimated average intake; GTN: Gauteng; FS: Free State; NW: North West; NFCS: National Food Consumption Survey.

**Figure 5:**
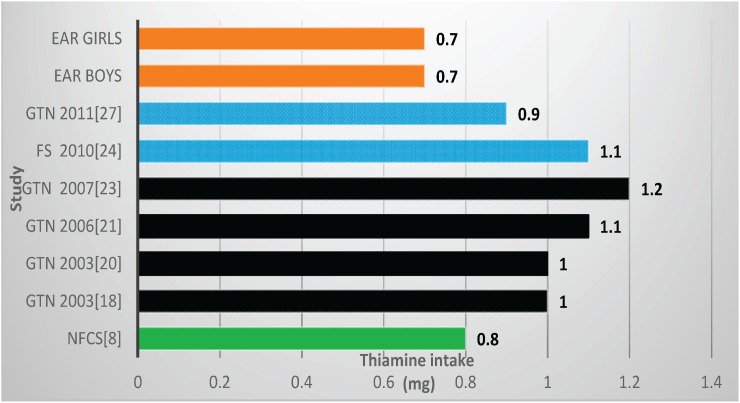
Mean thiamine intake in studies on schoolchildren in South Africa between 2000 and 2015 with the blue bars representing two studies which included fortification values. EAR: estimated average intake; GTN: Gauteng; FS: Free State; NW: North West; NFCS: National Food Consumption Survey.

Two studies reported mean zinc values below the EARs, both in Gauteng Province, with both including fortification nutrients [25,27] (Table 2, Figure 3). The NFCS also reported a mean value below the EAR. Four of the six studies (which reported vitamin A values) showed mean vitamin A intakes less than the EARs (Table 2, Figure 4). These include *two of the four* studies which had fortification nutrients added [24,27]. The NFCS had a mean vitamin A intake above the EARs. There were no studies that showed thiamine levels below the EARs (Figure 5). The NFCS mean vitamin A and thiamine values were above the EARs. 

**Figure 6:**
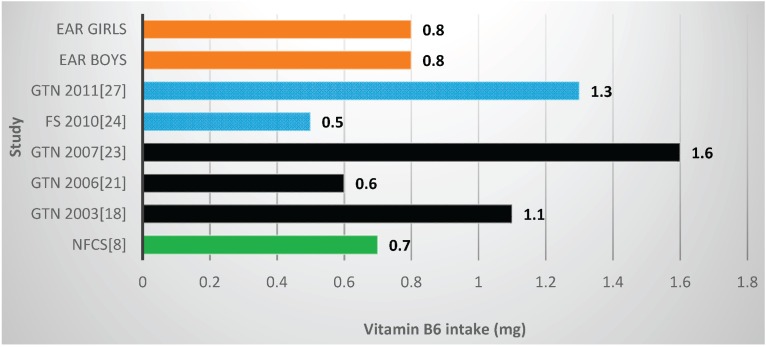
Mean vitamin B6 intake in studies on schoolchildren in South Africa between 2000 and 2015 with the blue bars representing two studies which included fortification values. EAR: estimated average intake; GTN: Gauteng; FS: Free State; NW: North West; NFCS: National Food Consumption Survey.

**Figure 7:**
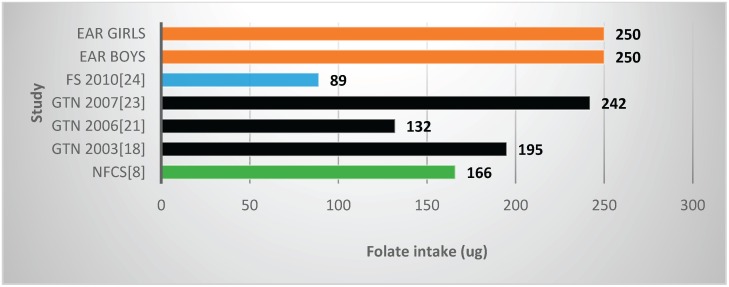
Mean folate intake in studies on schoolchildren in South Africa between 2000 and 2015 with the blue bars representing one study which included fortification values. EAR: estimated average intake; GTN: Gauteng; FS: Free State; NW: North West; NFCS: National Food Consumption Survey.

**Figure 8:**
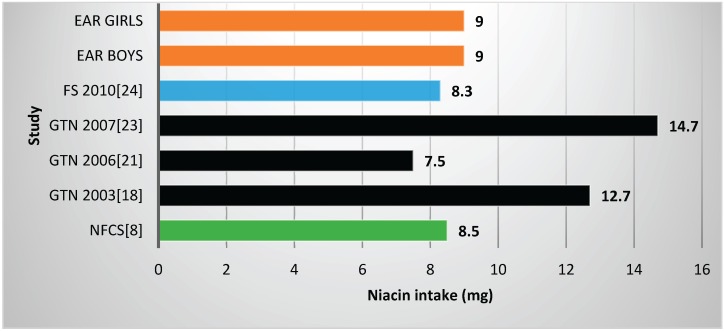
Mean niacin intake in studies on schoolchildren in South Africa between 2000 and 2015 with the blue bars representing one study which included fortification values. EAR: estimated average intake; GTN: Gauteng; FS: Free State; NW: North West; NFCS: National Food Consumption Survey.

**Figure 9:**
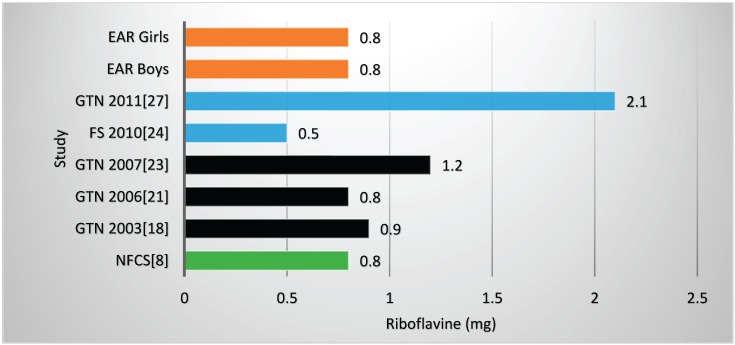
Mean riboflavin intake in studies on schoolchildren in South Africa between 2000 and 2015 with the blue bars representing two studies which included fortification values. EAR: estimated average intake; GTN: Gauteng; FS: Free State; NW: North West; NFCS: National Food Consumption Survey.

Two studies and the NFCS reported mean vitamin B6 values below the EARs (Table 2, Figure 6), one with fortification nutrients added [24] and one without [21]. Mean vitamin B6 value for the NFCS was below the EARs. All studies and the NFCS showed folate levels below the EARs (Table 2, Figure 7). Of the four studies which measured niacin, including one with fortification nutrients [24] and one without [21], two showed levels below the EAR values (Table 2, Figure 8). The mean value for the NFCS was less than the EARs for niacin. Only one study, which included fortification nutrients [24], reported a riboflavin value less than the EAR. The NFCS showed a mean riboflavin value above the EARs (Table 2, Figure 9). 

The foods commonly eaten as reported in five of the studies are shown in Table 3. There appear to be large differences between studies. In Limpopo Province [28] and in Free State Province [24] fortified maize was eaten by 98% of the participants, while this decreased to 34.5% in Gauteng Province [27]. Fortified bread was eaten by more than 90% of participants in Limpopo Province [28] and Gauteng Province, but only by 45% in Free State Province [24] and 64% in one study in Gauteng Province [25]. The amount of bread eaten varied between 85 g (three slices) to 146 g (five slices) per day, while the amount of maize porridge varied between 141 g (1/2 cup) to 628 g (21/2 cups) per day. These results confirm that the two fortified vehicles were eaten by children in all studies.

## 4. Discussion 

Our review showed that comprehensive dietary intake data that may reflect the national data are not available. Overall, there were 10 studies in schoolchildren post the NFCS of which one was in Free State Province [24], two were in North West Province [19,20] and the rest were in Gauteng Province [18,21,22,23,25,26,27]. The majority of studies reflect urban data for black subjects, and with the exception of the North West Province studies [19,20], which had small sample sizes making it difficult to generalize the data. The majority of studies in the review [18,21,23,24,25,26,27] published their results as means with boys and girls together. No explanation was given for this and this can be regarded as a further limitation regarding the generalizability of the data. This review clearly illustrates the importance of obtaining national representative data and is in itself an important finding for other countries in a similar situation since national representative intakes are important for policy makers in order to develop and implement health interventions. The intention of this review was to see if one could find sufficient studies representative of the different provinces to predict quantified nutrient intakes in lieu of an absence of national data. 

Only 5 of the 10 studies used more than one 24 HR or a QFFQ for dietary intake assessment. These methods better reflect usual intake than a single 24 HR [29,30]. However, the large sample sizes of two of the studies that used a single 24-h recall [19,20] may attenuate the limitations of this method. Micronutrient intake data presented in the Oldewage-Theron (2006) study [21] may underestimate the intake of all the fortification nutrients as these were not added as part of their nutrient intake quantification due to fortification data not added to food tables. It further needs to be considered that only three of the nine provinces in the country were represented in the identified studies. Provinces in South Africa vary considerably both geographically and economically, hence it is difficult to generalize data from one province to another. 

Mean energy intake was found to be below the EER in all studies, which could be expected to result in a high prevalence of underweight and low or no prevalence of overweight/obesity in this age group. Considering the fact that the documented underweight prevalence of 8.6% for boys and 4.0% for girls in the SANHANES [7] reflects “low prevalence” based on the WHO cut-off value of <10% for public health significance, as well as the fact that obesity prevalence in this age group has been increasing, the validity of the EER values should be questioned. Within the context of the mentioned anthropometric indicators, it is possible that under-reporting may have been a limitation in all studies, irrespective of the dietary methods used [29,30]. This limitation has bearing on the interpretation of the adequacy of intake of all other nutrients reported in the studies reviewed. The lowest energy intake was reported by Oldewage-Theron et al. for children in a very poor rural area called Qwa Qwa in Free State Province [24]. The low intakes in this study may be a true reflection of intake as three 24 HRs were used for dietary intake assessment, and may be associated with the poor socio-economic circumstances of these children. 

Considering the apparent inadequate energy intake, it is interesting to note that mean protein intakes were above the EARs in all studies, even in the Free State Province study [24]. As no detail on the sources of protein (i.e. plant versus animal) and actual food choices were reported, it is not possible to speculate on this finding of our review. However, if the typical dietary patterns of South Africans are considered, especially black South Africans in urban areas, the intake of legumes [8], could have contributed to protein intakes.

With fortification in place since 2004, the expectation would be for any dietary inadequacies in terms of micronutrient intakes that were reported prior to fortification by Labadarios et al. [8] in the NFCS), Mackeown et al. [18], Schutte et al. [19,20], Peterson et al. [22], and MacKeown et al. [23], would have been addressed. However, review of micro-nutrient intakes reported in the studies included in this review, including the NFCS [8], firstly shows that mean intake of fortification nutrients were not necessarily below the EAR before fortification. Secondly, results do not reflect complete resolution of the purported micronutrient intake risks post fortification. It needs to be mentioned that risk of deficiency for the purposes of this review was defined as a mean intake below the EAR, whereas the NFCS used a cut-off of intake below 67% of the RDA, which may have resulted in overestimation of risk of deficiency in that study. It is important to note that the micronutrients selected for fortification were based on the results of the NFCS (thus based on the higher cut-off).

When comparing micronutrient intake results across studies, it is important to bear in mind that there were three types of studies. Firstly, studies which were undertaken before fortification became mandatory, namely prior to 2004; secondly, studies which took place after 2004 but the researchers did not include the fortification in their data analyses; and thirdly, studies which took place after fortification and where the researchers did include the fortification nutrients in their analyses. Unfortunately, only four studies [24,25,26,27] reported on micronutrient intakes and in some instances not all the relevant ones.

Mean iron intakes were reported on in seven of the reviewed studies [18,19,20,21,23,24,27], as well as the NFCS [8]. Results reflect no iron deficiency risk in all, including the NFCS, but one of these studies, the latter being the Free State Province study [24]. This implies that iron intakes were adequate pre- and post-fortification, despite the possibility that underestimation may have been a limitation in most of the studies. Data from the SANHANES study in 2012 support a low prevalence of iron deficiency since only 10.5% of 16–25 years old adolescents and women were found to have iron deficiency anemia (Hb ≤ 12 g/dL and ferritin ≤ 15 μg/L These results that point to adequate intakes prior to 2006 are not aligned with the estimation of iron deficiency anemia in South African children and adolescents of 25.5% by the Global Burden of Disease 2013 Study [31]. A possible explanation for this situation is that iron intake in the investigated communities may be mostly in the non-heme form, with poor bioavailability thus contributing to iron undernutrition found in children and adolescents, despite adequate intakes. It needs to be seen whether the seemingly adequate iron intakes reported in the post fortification era will result in improved iron nutriture in the study areas and the country as a whole in the years to come. 

Mean zinc intakes were reported on in six of the reviewed studies [18,19,21,23,25,27]. The mean intake reported in the NFCS was below the EAR, reflecting actual risk of zinc deficiency. Inadequate post fortification (2010) intakes were reported by Samual et al. [25] for urban blacks (despite fortification nutrients being included in quantification of intake) and by Oldewage-Theron et al. [21], who reported on post-fortification (2006) for another group of urban blacks living in informal settlements in Gauteng Province. Fortification with nutrients was not included in quantification of intake of the latter study, thus reported low intakes may thus be the result of underestimation of actual intake. As there is a paucity of information on the actual zinc status of South African children and adolescents, the validity of these dietary intake results need further investigation.

Mean vitamin A intakes were reported on in six of the reviewed studies [18,19,21,23,24,27], including the Free State Province study [24], as well as the NFCS [8]. The mean intake found in the NFCS was above the EAR, thus reflecting no vitamin A deficiency risk, as opposed to the interpretation of the NFCS using the <67% RDA cut-off, which indicated risk [8]. Only two other studies conducted in Gauteng Province [21,23] reported adequate intakes. Data on the blood vitamin A status of female adolescents and adults (16–25 years) who participated in SANHANES [7] showed that 11.6% had a mild vitamin A deficiency of <0.7 μmol/L.

As for B-vitamin intakes, it is interesting to note that the NFCS mean nutrient intakes were below the EARs only for niacin, folate, and vitamin B6, but not for thiamine and riboflavin, indicating that fortification with the latter two vitamins may not have been necessary. The other two pre-fortification studies and all of the post-fortification studies showed that thiamine intakes were above the EAR. 

There were two studies that consistently showed the lowest micronutrient intakes, with the exception of thiamine. These included a study by Oldewage-Theron et al. [21] in Gauteng Province in an informal settlement with dietary intake measured by means of a QFFQ and another study by the same author where dietary intake was measured by means of three 24 HRs in Free State Province [24]. It needs to be noted that the dietary methodology used in both these studies have been reported to provide a better indication of usual intake than for example the single 24 HR used in some of the other studies included in this review. Further support for the possibility that intakes in these two areas were truly low comes from the fact that Qwa Qwa in Free State Province is a very poor rural area and intakes can thus be expected to be low [24]. The informal settlement, previously called a squatter camp, in Gauteng Province is also characterized by having participants from very low socio-economic strata, with many being unemployed [21]. However, since the fortification nutrients were not added to the dietary data in the latter study [21] it is likely that the micronutrient results presented by these researchers are underestimated. Micronutrient intake data reported by Schutte [19], Schutte [20], and Oosthuizen [27] need to be interpreted with caution as the dietary method used was a single 24 HR. The same is true for the NFCS, although the sample size in the latter does increase the validity of the outcomes. 

With mandatory fortification, the expectation was that all the deficient nutrients would be met. However, based on the finding that there were only four studies which included the fortification nutrients [24,25,26,27], one is unable to say with some assurance that fortification of maize and bread have improved micronutrient intakes. The study in Free State Province [24] was done in a very poor area and it is possible that even fortification of staple foods was not sufficient to improve low intake of micronutrients which would also have been affected by the low energy intakes. However, another study, which included fortification, was done in a peri-urban area of Gauteng Province in 2011 [27] where dietary intakes patterns of the children were based on carbohydrates and fat with a low consumption of fruit and vegetables. The latter study showed significantly higher intakes of mean vitamin B6, riboflavin, zinc, and iron when compared with the NCHS results of 1999. These findings do suggest that fortification was responsible for the higher intakes in this study. Furthermore, a study was undertaken in 2008 which calculated the theoretical mean intakes of micronutrients after addition of the fortificants to the diets of the NFCS children [12]. Significant improvements were found in intakes of all the micronutrients, particularly in children living in the poorer rural areas.

Food intake data from the present review shows that in studies which examined the percentages of schoolchildren who consumed fortified bread or maize meal or both, intakes were high, indicating that one or both fortified foods were consumed by nearly everyone. This is a very positive finding, since it indicates that the vehicles used for fortification are correct.

In summary, the benefits of this review are the following: Firstly, a number of studies presented data on macro- and micronutrient intakes which can be compared with those of schoolchildren in other low and middle income countries. Secondly, the importance of having up to date food composition tables is critical for doing dietary intake studies. In this study, it is shown that the food composition tables were not updated to reflect food fortification values after mandatory fortification was introduced. This makes a significant difference to the actual values of specific micronutrients which were reflected in the various studies. Thirdly, this study illustrates the importance of doing a national study to obtain dietary intakes and, furthermore, it shows that if such funding is not available one can still use the data from local studies to make a reasonable assessment of the situation, which is better than having no data available. Such data is essential if governments wish to fortify specific foods, since they need to determine which nutrients are deficient in the diet.

## 5. Conclusions 

Overall, there is a paucity of dietary studies which have included fortified nutrients, making it difficult to determine whether fortification has improved the micronutrient intakes of schoolchildren. This is further complicated by the fact that different dietary methods were used and that studies were only done in three of the nine provinces. The results of these studies clearly point to the importance of doing a national study on the dietary intake of schoolchildren in order to confirm the outcomes of the fortification process.

## Figures and Tables

**Table 1:** Dietary intake studies from 2000 onwards that included daily energy and protein intake data from children between the ages of 6 and 15 years.

Authors	Pub. Year	Age (Years)	Province	Location & Ethnicity	N	Dietary Method	Energy Intake (KJ) (Mean and SD or CI)	Protein Intake (g) (Mean and SD or CI)
^1^ Labadarios et al. [8] ^$^	2000	7–9	National	All	477	24 HR	5800 (3988–6149)	35 (23)
MacKeown et al. [18] ^#^	2003	10	Gauteng	UB	163	QFFQ	Boys & girls 7323 (7043–7603)	55 (53–58)
^1^ Schutte, et al. [19]	2003	10–15	North West	UB & RB	694	24 HR	Boys: 7822 (3184); Girls:7224 (2532)	Not given
^1^ Schutte, et al. [20]	2003	10–15	North West	All groups	1244	24 HR	Not given	B & C: 61 (0.8); I & W: 64 (1.6)
^2^ Oldewage-Theron et al. [21]	2006	9–13	Gauteng	UB	149	QFFQ	Boys & girls: 5990 (392)	39.6 (20.4)
^1^ Peterson et al. [22] ^#^	2006	11	Gauteng	UB & UW	202	24 HR	Girls: B 5422 (2242); W: 5055 (1784)	Not given
^1^ MacKeown et al. [23] ^#^	2007	13	Gauteng	UB	143	QFFQ	Boys & girls: 8912 (8355–9469)	59 (55–63)
^3^ Oldewage-Theron & Egal [24]	2010	9–13	Free State	RB	142	3 × 24 HR	Boys & girls: 4309 (1410)	37 (13)
^3^ Samuel et al. [25]	2010	7–11	Gauteng	UB	149	2 × 24 HR	Boys & girls: 5121 (2399)	37.8 (19)
^3^ Oldewage-Theron et al. [26]	2011	6–13	Gauteng	UB	97	24 HR	Boys & girls: 4943 (2362)	37 (18)
^3^ Oosthuizen et al. [27]	2011	9–13	Gauteng	UB	75	24 HR	Boys & girls: I group 6545; C group 6324	52.2 and 54.7

^1^ Data collection pre-fortification; ^2^ Data collection post-fortification but fortification nutrients not added in dietary intake quantification; ^3^ Data collection post-fortification; fortification nutrients were added in dietary intake quantification; ^$^ National food consumption survey undertaken in 1999; ^#^ Results from BIRTH2TWENTYcohort; Pub. = publication date; R = rural; U = Urban; B = black; W = white; 24 HR = 24 h recall; QFFQ = quantified food frequency; SD = standard deviation; CI = 95% confidence interval; I = intervention; C = control.

**Table 2:** Dietary intake studies from 2000 onwards that included daily micronutrient data from children between the ages of 6 and 15 years.

Authors	Iron (g) Mean SD/CI	Zinc (mg) Mean SD/CI	Vitamin A (μg) Mean SD/CI	Thiamine (mg) Mean SD/CI	Vitamin B6 (mg) Mean SD/CI	Folate (μg) Mean SD/CI	Niacin (mg) Mean SD/CI	Riboflavin (mg) Mean SD/CI
^1^ Labadarios et al. [8] ^$^	6.9 (5.2)	5.8 (3.8)	493 (1328)	0.8 (0.4)	0.7 (0.5)	166 (140)	8.5 (6.2)	0.8 (1.0)
^1^ MacKeown et al. [18] ^#^	8.5 (8.1–8.9)	7.7 (7.4–8.1)	324 (301–348)	1.0 (0.9–1.1)	1.1 (1.0–1.2)	195 (186–205)	12.7 (12–13)	0.9 (0.8–0.9)
^1^ Schutte, et al. [19]	7.9 (3.8) to 8.9 (4.6)	7.4 (4) to 8.9 (4.6)	369 (613)–467 (701)	Not given	Not given	Not given	Not given	Not given
^1^ Schutte, et al. [20]	8.4 (0.2) to 8.7 (0.3)	Not given	Not given	Not given	Not given	Not given	Not given	Not given
^2^ Oldewage-Theron [21]	5.8 (4.3)	4.9 (3.0)	460 (430)	1.1 (0.4)	0.6 (0.7)	132 (121)	7.5 (6.0)	0.8 (0.9)
^1^ Peterson et al. [22] ^#^	Not given	Not given	Not given	Not given	Not given	Not given	Not given	Not given
^1^ MacKeown et al.[23] ^#^	10.2 (9.5–11.0)	8.2 (7.7–8.8)	620 (546–694)	1.2 (1.1–1.2)	13 years: 1.6 (1.5–1.7)	242 (223–261)	14.7 (13.6–15.8)	1.2 (1.1–1.3)
^3^ Oldewage-Theron [24]	5.3 (1.9)	Not given	220 (87)	1.1 (0.4)	0.5 (0.6)	89.2 (78.9)	8.3 (4.7)	0.5 (0.3)
^3^ Samuel, et al. [25]	Not given	4.6 (2.2)	Not given	Not given	Not given	Not given	Not given	Not given
^3^ Oldewage-Theron [26]	Not given	Not given	Not given	Not given	Not given	Not given	Not given	Not given
^3^ Oosthuizn et al. [27]	I = 10.6; C = 11.8	I = 7.4; C = 9.0	I = 336; C = 682	I = 0.9; C = 1.1	I = 1.3; C = 1.3	Not given	Not given	E = 2.1; C = 2.2

^1^ Data collection pre-fortification; ^2^ Data collection post-fortification but fortification nutrients not added in dietary intake quantification; ^3^ Data collection post-fortification; fortification nutrients were added in dietary intake quantification; ^$^ National food consumption survey undertaken in 1999; ^#^ Results from BIRTH2TWENTYcohort; Pub. = publication date; R = rural; U = Urban; B = black; W = white; 24 HR = 24 h recall; QFFQ = quantified food frequency questionnaire; SD = standard deviation; CI = 95% confidence interval; I = intervention; C = control.

**Table 3:** Percentage of schoolchildren who consumed a fortified item on the previous day/days in four studies which reported frequency of consumption in the post-fortification era.

Fortified Foods Eaten Dietary Method	^1^ Limpopo 2007 [28] 1 × 24 HR	Free State 2010 [24] 3 × 24 HR	Gauteng 2011 [27] 1 × 24 HR	Gauteng 2010 [25] 2 × 24 HR
Age of children	10–13 years	9–13 years	9–13 years	7–11 years
Fortified maize meal (frequency)	98.1%	99.3%	34.5%	92%
Amount maize meal (g)	Not given	402	141	628
Fortified bread (frequency)	98.7%	45.1%	94.5%	64%
Amount bread consumed (g)	Not given	113	146	85

^1^ Limpopo study was not part of the 10 studies since only foods were reported and not nutrients.

## Abbreviations

The following abbreviations are used in this manuscript:

NFCS	National food consumption survey
EAR	Estimated adequate intake
RDA	Recommended dietary allowance
24 HR	24 h recall
QFFQ	Quantified food frequency questionnaire
